# The Genetic Characterization of the First Detected Bat Coronaviruses in Poland Revealed SARS-Related Types and Alphacoronaviruses

**DOI:** 10.3390/v14091914

**Published:** 2022-08-30

**Authors:** Anna Orłowska, Marcin Smreczak, Katarzyna Thor, Magda Niedbalska, Dominika Pawelec, Paweł Trębas, Jerzy Rola

**Affiliations:** 1 Department of Virology, National Veterinary Research Institute, 24-100 Puławy, Poland; 2 Department of Animal Genetics and Conservation, Institute of Animal Sciences, Warsaw University of Life Sciences—SGGW, 02-786 Warsaw, Poland

**Keywords:** coronaviruses, bats, SARS-related CoV, prevalence, Poland, phylogenetics

## Abstract

Bats are a major global reservoir of alphacoronaviruses (alphaCoVs) and betaCoVs. Attempts to discover the causative agents of COVID-19 and SARS have revealed horseshoe bats (Rhinolophidae) to be the most probable source of the virus. We report the first detection of bat coronaviruses (BtCoVs) in insectivorous bats in Poland and highlight SARS-related coronaviruses found in Rhinolophidae bats. The study included 503 (397 oral swabs and 106 fecal) samples collected from 20 bat species. Genetically diverse BtCoVs (*n* = 20) of the *Alpha*- and *Betacoronavirus* genera were found in fecal samples of two bat species. SARS-related CoVs were in 18 out of 58 lesser horseshoe bat (*Rhinolophus hipposideros)* samples (31%, 95% CI 20.6–43.8), and alphaCoVs were in 2 out of 55 Daubenton’s bat (*Myotis daubentonii*) samples (3.6%, 95% CI 0.6–12.3). The overall BtCoV prevalence was 4.0% (95% CI 2.6–6.1). High identity was determined for BtCoVs isolated from European *M. daubentonii* and *R. hipposideros* bats. The detection of SARS-related and alphaCoVs in Polish bats with high phylogenetic relatedness to reference BtCoVs isolated in different European countries but from the same species confirms their high host restriction. Our data elucidate the molecular epidemiology, prevalence, and geographic distribution of coronaviruses and particularly SARS-related types in the bat population.

## 1. Introduction

Wild animals represent a large and little-explored reservoir that plays a key role in the emergence of previously unknown pathogens which pose a significant threat to human and domestic animal welfare. Bats (Chiroptera) are the most numerous and diverse mammals worldwide and are the only terrestrial mammals with the abilities to fly in the true sense and use echolocation. With over 1400 species, bats are the second largest group of mammals after rodents [1,2]. In recent years, bats have been recognized as a reservoir of numerous emerging viruses such Ebola virus, Nipah virus, and coronaviruses, which can cross the species barrier and infect humans and animals [3,4,5]. Studies conducted by different groups of scientists have proven that bats serve as a major reservoir of alphacoronaviruses (alphaCoVs) and betaCoVs. It has also been posited that bats may harbor an ancestor for both CoV genera [6,7].

The COVID-19 pandemic has prompted a worldwide search for the source of the SARS-CoV-2 virus and the other members of the *Sarbecovirus* subgenus. The zoonotic nature of SARS-CoV-2 is widely accepted within the scientific community, but its animal reservoir remains unidentified. Horseshoe bats (Rhinolophidae) have been reported to harbor the virus most genetically similar to the SARS-CoV-2 responsible for the pandemic, which is believed to have passed through an unknown intermediate host before spilling over into the human population [8,9,10]. One bat coronavirus (BtCoV) discovered in a cave-dwelling Rhinolophidae bat in China’s Yunnan Province in 2013 revealed 96.2% nucleotide sequence similarity with a SARS-CoV-2 reference strain detected in November 2019 in Wuhan [11]. Phylogenetic analyses of novel horseshoe bat sarbecoviruses in China have shown these to be most closely related to both SARS-CoV and SARS-CoV-2, and a coronavirus recently reported from a species of horseshoe bat in Thailand was determined to be a SARS-CoV-2-like virus [12].

Since the onset of the SARS epidemic, more than 35 alphaCoVs and betaCoVs have been detected in various bat species of the Yinpterochiroptera and Yangochiroptera suborders worldwide, and evidence of infection with coronaviruses has been demonstrated in 11 of the 18 frugivorous or insectivorous bat families [13,14]. The majority of BtCoVs were reported in insectivorous kinds and only four species were noted in frugivorous bats. Until now in Europe, coronaviruses in bats have been reported in several countries including Germany, Denmark, Italy, France, Switzerland, Hungary, Finland, Slovenia, and Luxembourg with prevalences between 4.2 and 21.4%. Bat coronaviruses were detected in numerous species such as *Myotis* bats (*M. daubentonii*, *M. brandtii*, *M.* *dasycneme*, *M. myotis*, *M. nattereri*, *M. alcathoe*, *M. oxygnathus* and *M. blythii*), Serotine bats (*Eptesicus nilssonii*), Pipistrelle bats (*P. nathusii*, *P. pygmaeus*, *P. pipistrellus*, *P. kuhlii*, *P. auritus*), *Rhinolophus ferrumequinum*, *Hypsugo savii,* and *Nyctalus noctula* [15,16,17,18,19,20,21,22,23,24,25,26,27]. SARS-related coronaviruses, however, were detected mainly in horseshoe bats which are the main reservoirs of the viruses, the majority of such detections were in Asia but some were in Europe and Africa. In Europe, evidence of bat infection with SARS-related coronaviruses was found in Hungary, Italy, Slovenia, Spain, Bulgaria, and the UK [18,28,29,30,31]. Although several studies have been conducted on bats, information is still incomplete on the host specificity of coronaviruses in bats and on which genera and strains of BtCoVs are circulating in Europe. In view of the ongoing COVID-19 pandemic and the possibility of species jumping (bat–human or human–bat), coronavirus elucidation is particularly important for animal and public health. Therefore, it is essential to monitor the circulation of coronaviruses including SARS-CoV-2 in the field.

Here, we present the first report of the detection of CoVs in the population of different bat species in Poland, where a salient detection is in a Rhinolophidae bat which is considered the natural reservoir of SARS-related CoVs. The study was focused mainly on identifying sarbecoviruses as contributive work for determining the natural reservoir of SARS-CoV-2 and to better understand the genomic evolutionary structure of European BtCoVs. We were also able to detect the CoVs using a pan-corona nested PCR procedure and thereby were able to study the prevalence and genetic diversity of BtCoVs circulating in bats in Poland. This is the first report on BtCoV prevalence in Poland. The paper also provides new data on the presence of sarbecoviruses in Europe and other alphaCoVs. It also includes findings for the genetic diversity of BtCoVs which may be crucial for the understanding of basic evolutionary patterns of European BtCoVs and interspecies transmission.

## 2. Materials and Methods

### 2.1. Samples

A total of 503 samples (397 oral swabs and 106 fecal) were collected from bats roosting in Poland between May and September of 2020–2022. At these summer sites, samples were collected from bats of both sexes, including individuals belonging to 20 species and 38 bats for which the species was not identified (Table 1). Samples were collected by qualified chiropterologists from bats captured for fauna surveys quantifying bat populations. Swabs were collected by swabbing the oral cavity of bats with a Copan Universal Transport (UTM-RT) System (Copan, Brescia, Italy). Fecal pellets were collected into a sterile 1.5 mL microcentrifuge tube and kept at 4 °C until delivery to the laboratory. In addition to samples from individual bats, guano samples were collected from bat roosting sites. In the laboratory, oral swabs and feces samples were kept frozen at −80 °C until RNA extraction. Trapping and biometric measurements were performed with permission from the General Directorate of Environmental Protection in Warsaw. Bat species and sex were identified morphologically by the appearance of characteristics and genitalia.

### 2.2. RNA Extraction

Total RNA was extracted from 200 μL of oral swabs and 100 μL of 10% (*w*/*v*) fecal homogenate centrifuged at 14,000 rpm (16,000× *g*) for 3 min. The procedure used an IndiMaq Pathogen Kit (Indical Biosciences Gmbh, Leipzig, Germany) and involved automated extraction of viral RNA/DNA with an Indical magnetic particle processor. Nucleic acids were eluted in 100 μL AVE buffer and immediately subjected to BtCoV detection or kept frozen at −80 °C for further investigation.

### 2.3. Molecular Detection of BtCoVs and Sequencing

The extracted RNA was first screened for the presence of sarbecoviruses using a real time RT-PCR (rtRT-qPCR) method previously described by Corman based on detection of the E gene of sarbecoviruses [32]. Amplicons with positive results were subjected to Sanger sequencing. Subsequently, total RNA from bat samples was investigated to detect other CoVs using the method of De Souza-Luna et al. [33] with modifications. Reverse transcription was performed in a 20 μL volume mixture containing 50 ng of random hexamers and Superscript III Reverse Transcriptase (Thermo Fisher Scientific, Waltham, MA, USA) using the manufacturer’s instructions. The most conserved region of the RNA-dependent RNA polymerase (RdRp) gene was the target of a nested reverse transcriptase-PCR (nRT-PCR) for BtCoV screening. Briefly, 1 μL of cDNA was added to a reaction mixture containing 0.1 mM dNTP, 2.5 mM MgCl2, PlatinumTaq DNA polymerase, and a primer mix of 20 μM each. In the next step, 1 μL of PCR product was used for nRT-PCR following the protocol of De Souza-Luna et al. referred to previously [33]. Amplification was performed in a ProFlex thermocycler (Thermo Fisher Scientific) with the following program: 1 cycle at 95 °C for 5 min followed by 40 cycles at 94 °C for 20 s, at 60 °C for 30 s, and at 72 °C for 1 min and a final elongation at 72 °C for 10 min. Amplicons were detected by separation on 1% agarose gel and after purification were sequenced in both directions. Sanger sequencing of both rtRT-qPCR and nRT-PCR amplicons was performed with automation on an ABI PRISM 310 Genetic Analyzer using a BigDye Sequencing Kit with GeneScan Analysis Software (all from Applied Biosystems, now Thermo Fisher Scientific).

### 2.4. Phylogenetic Analysis

Nucleotide sequences of 440 bp of the RdRp regions of Polish BtCoVs were aligned using Clustal W Multiple alignment 7.0.5.3. A similarity matrix was constructed using BLOSUM62 in BioEdit software v. 7.0.5.3. A phylogenetic tree was generated using the neighbor-joining method with the Kimura-2parameter evolutionary model and bootstrapped on a set of 1000 replicates with MEGA software version 5 [34]. To determine the phylogenetic relationship of coronaviruses isolated from bat samples collected in Poland, five nucleotide sequences of BtCoVs were compared to reference sequences (available in the GenBank database) guided by the closest phylogenetic relationships and geographical criteria.

## 3. Results

### 3.1. Prevalence of BtCoVs in Different Bat Species in Poland

**Figure 1:**
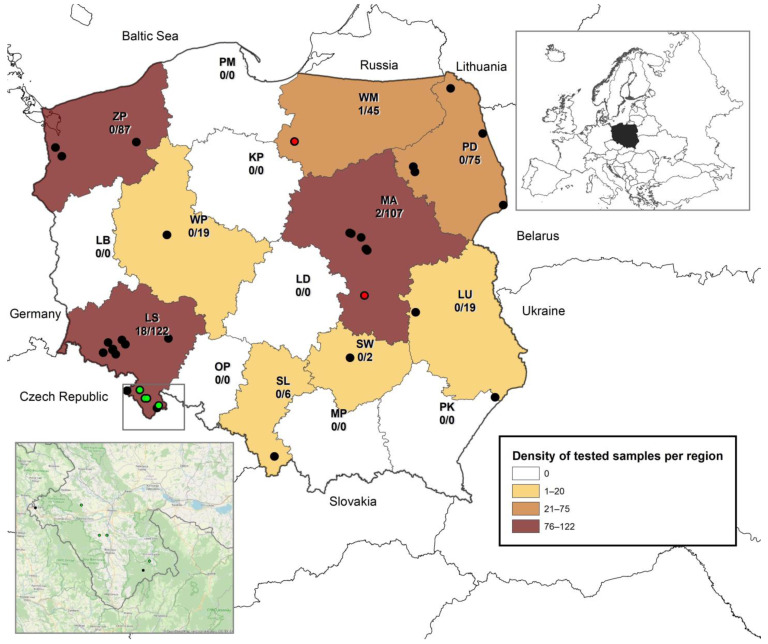
Map showing the voivodeships of Poland and the origin of bat samples included in the study with their RT-PCR results (black: negative; red: positive (alphaCoV); green: positive (SARS-related CoV)). The number of positive vs. total samples per region is indicated. AlphaCoVs were detected in bat feces collected in June 2020 at Warmian-Masurian (WM, accession number ON873766) and in September 2021 at Mazovian (MA, accession number ON873768). SARS-related CoVs were detected in bat samples collected in May 2021 and May 2022 at Lower Silesia (LS), accession numbers: ON873767, ON873769, and ON873770. Abbreviations: MA: Mazovian; LS: Lower Silesia; WP: Greater Poland; SL: Silesia; PM: Pomerania; LD: Łódź; MP: Lesser Poland; ZP: West Pomerania; LB: Lubusz; KP: Kuyavian-Pomeranian; OP: Opole; PD: Podlaskie; SW: Świętokrzyskie; WM: Warmian-Masurian; PK: Subcarpathian; LU: Lublin.

A total of 503 bat samples divided almost equally between the sexes, originating from 9 out of the total 16 voivodeships were tested for the presence of BtCoV RNA (Figure 1). Geographical location was not available for 21 samples. The largest numbers of samples originated from the Mazovian, Lower Silesia, West Pomeranian, and Podlaskie voivodeships (*n* = 75–122). Samples from the Warmian-Masurian voivodeship comprised a smaller proportion (*n* = 45). Twofold fewer samples than from the Warmian-Masurian voivodeship were collected from Greater Poland and Lublin (*n* = 19), and only a low number of samples originated from Świętokrzyskie and Silesia voivodeships (*n* = 2–6) (Figure 1). *Nyctalus noctula* comprised the largest proportion for one species of bat (*n* = 79; 15.7%) followed by *Pipistrellus* spp. (*n* = 64; 12.72%), *Rhinolophus hipposideros* (*n* = 58; 11.53%), *Eptesicus serotinus* (*n* = 57; 11.33%), *Barbastella barbastellus* and *Myotis daubentonii* (each *n* = 55; 10.93%), and *Plecotus auritus* (*n* = 30; 5.96%). Other indigenous bat species were sparsely represented (Table 1). For 38 samples (7.55%), bat species identification failed. The majority of unidentified samples consisted of guano samples collected from maternity colonies. Since there was no 100% certainty of bat origin, moreover, no BtCoVs RNA was detected in all unidentified samples, they were included in the paper as unidentified.

The detection of sarbecoviruses with rtRT-qPCR obtained positive results with Ct values ranging between 21.3 and 37.5 for 18 out of a total of 503 tested samples (3.6%, 95% CI 2.3–5.6). RNA of 18 sarbecoviruses was detected in samples collected in four roosts of lesser horseshoe bats (*Rhinolophus hipposideros*) in Lower Silesia. The further investigation performed for the all 503 bat samples using nested pan-coronavirus primers additionally detected BtCoV in two fecal samples collected from *Myotis* bats (*M. daubentonii*) in two roosts in the Mazovian and Warmian-Masurian voivodeships, located over 230 km from each other. The samples positive for BtCoV RNA included 14 individual oral swabs and 6 guano samples and all positives were collected from female bats in May in the years 2020–2022 in maternity roosts. The overall prevalence of BtCoVs was estimated at 4.0% (95% CI 2.6–6.1), whereas for lesser horseshoe bats it reached 31.0% (95% CI 20.6–43.8). For *Myotis* bats the prevalence was 3.68% (95% CI 0.6–12.3). Table 1 summarizes the bat species from which the samples used in the study were collected and presents the prevalence rate of BtCoVs in individual species.

### 3.2. Phylogenetic Resemblance of Polish BtCoVs

**Figure 2:**
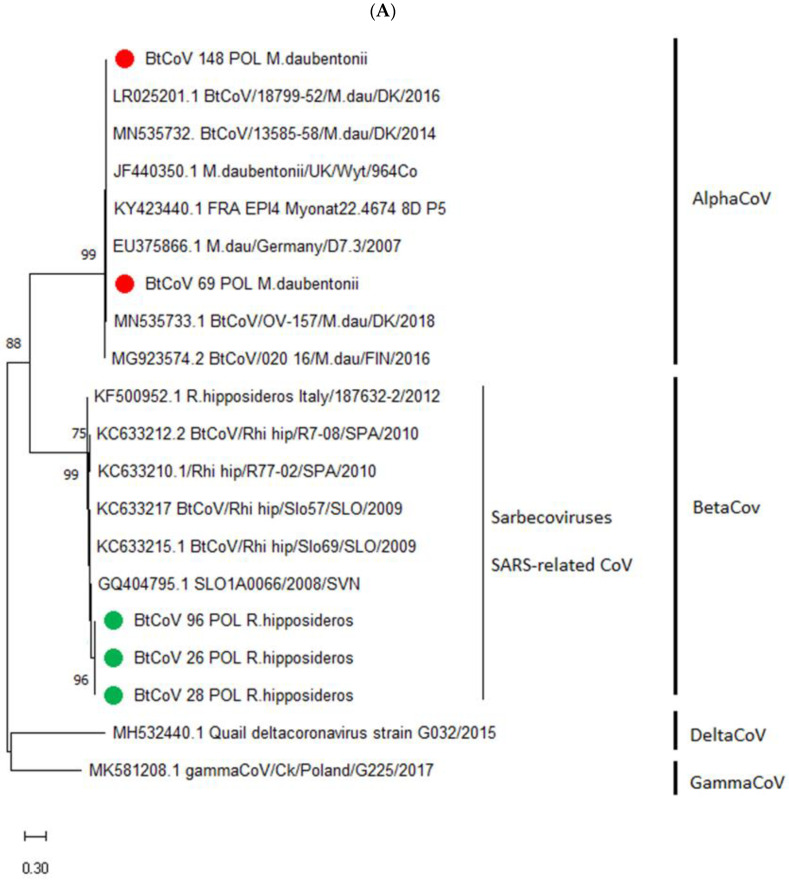

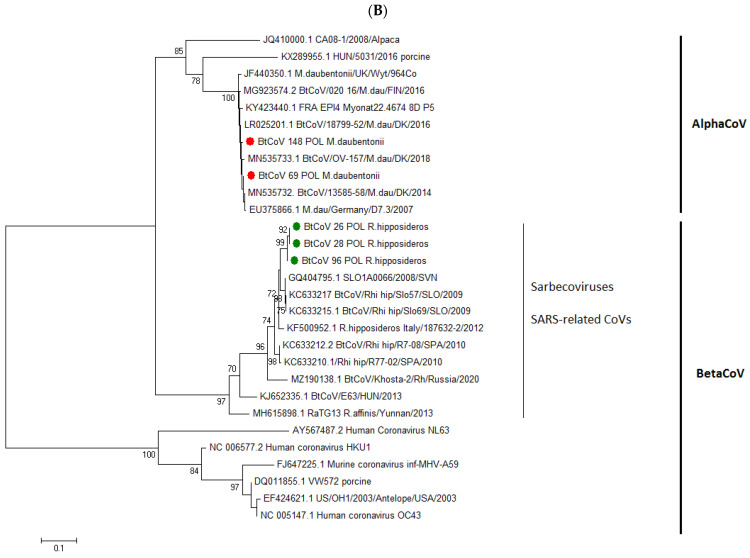
Phylogenetic tree based on the 379 bp-long nucleotide fragment of the RNA-dependent RNA polymerase gene of BtCoVs selected as the most homologous (**A**) and BtCoVs detected in Poland and the other distinct CoVs (**B**), generated using the neighbor-joining method with the Kimura2-parameter model and MEGA 5 software. Green circles indicate SARS-related CoVs detected in horseshoe bats in Poland, whereas red circles refer to alphacoronaviruses detected in *Myotis* bats in Poland. Bootstrap values (1000 replicates) over 70% indicating significant support for the tree topology are shown next to the branches. Sequences of delta- and gamma CoVs isolated from poultry in Poland were used as the outgroup in Figure 2A.

Coronavirus sequences of the RdRp gene 379 bp long were obtained for the 5 positive fecal samples with the highest viral loads, which were 3 from Rhinolophidae bats and 2 from *Myotis* species. The CoV sequences identified in horseshoe bats affiliated to the *Betacoronavirus* genus and *Sarbecovirus* subgenus and were determined as SARS-related coronaviruses by a BLASTn search and phylogenetic analysis comparison to reference CoV isolates. The sequence identity between BtCoVs identified in horseshoe bats in this study was estimated at between 99.2% and 100%. The sequences were submitted to GenBank and given accession Nos. ON873767, ON873769, and ON873770. Phylogenetic analysis performed for a 379 bp nucleotide sequence revealed the highest relationship to the SLO1A0066/2008/SVN isolate of bat coronavirus obtained in Slovenia from a fecal sample from a horseshoe bat in 2008, to which it had an identity of 96.3–96.5%. The next highest identity was to Spanish and Italian CoVs also isolated from horseshoe bats. The identity that ranged between 82.0 and 82.3% was estimated with the coronavirus RaTG13 strain detected in Yunnan province in China in 2013, suggested as a highly homologous prototype of SARS-CoV-2. Coronavirus sequences isolated from *Myotis* bats clustered with *Alphacoronavirus* genus isolates, and the highest nucleotide sequence identity of 99.2–99.4% was to BtCoV/13585-58/M.dau/DK/2014 detected in Denmark in a fecal sample from *Myotis daubentonii* in 2014. Other sequences to which these obtained in the present study had high identity were CoVs isolated from *Myotis daubentonii* bats in Finland, Germany, the UK, and France. These sequences were also submitted to GenBank and are registered under accession Nos. ON873766 and ON873768. Almost 99% identity was determined between alphacoronaviruses isolated from bats of this species in Poland even though they were collected over 230 km from each other. The phylogenetic relationships of the BtCoV isolated during this study to the reference CoV strains are presented in Figure 2.

Our study supported the host restriction specificity of certain CoV strains which was observed previously. SARS-related coronaviruses detected in Poland were obtained as other SARS-like viruses that were from horseshoe bats, whereas alphacoronaviruses detected in the Mazovian and Warmian-Masurian voivodeships from Daubenton’s bats in 2020 clustered on the phylogenetic tree with other European coronaviruses likewise detected from *Myotis daubentonii*.

## 4. Discussion

Numerous human and animal disease outbreaks in recent decades have identified bats as the source of various pathogens that pose a threat to both animal and public health on an ongoing basis. For the first two relevant epidemics of the 21st century, SARS in Asia and MERS in the Middle East, bat-borne transmission of coronaviruses was confirmed. The etiological agent of the current COVID-19 pandemic, SARS-CoV-2, likely originated from horseshoe bats, based on the over 96% identity to a SARS-related coronavirus isolated in a rhinolophid bat in 2013 in Yunnan province, China. Since the SARS epidemic in 2003 and the beginning of the intensive search for novel CoVs in various mammals, over 500 new CoVs have been identified in bats [35]. Some of the SARS-related CoVs identified in horseshoe bats in China easily replicated in human cells because they use angiotensin-converting enzyme 2 (ACE2) as their main cellular receptor which is present in both humans and bats [36]. Five of the seven human CoVs detected so far, i.e., the HCoVs-229E and HCoVs-NL63 alphacoronaviruses and SARS-CoV, MERS-CoV, and SARS-CoV-2, are strongly suggested to have been transmitted to humans by intermediate hosts [4]. Phylogenetic reconstructions for bat alpha-CoVs and bat beta-CoVs suggested an evolutionary origin of viruses within rhinolophid and vespertilionid bats [37]. It is supported by the fact that their genetic diversity is greater in bats than is currently known for any other host [13].

In the terms of the current pandemic, few studies have been conducted to detect sarbecoviruses in bat populations [38,39]. In this study, we investigated the presence of sarbecoviruses and the other CoVs in oral swabs and fecal samples from bat populations of different species and in different regions of Poland, investigating horseshoe bats most closely as the major reservoir of SARS-related coronaviruses. We applied the method of Corman et al. [32] routinely used in Poland for SARS-CoV-2 monitoring in mustelids. Subsequently, a nested RT-PCR procedure using pan-coronavirus primers was performed for CoVs detection. The study revealed the circulation of sarbecoviruses in Poland and confirmed the occurrence of infection with other CoVs in the Polish bat population. No studies on CoV prevalence in bat populations in Poland had ever been performed before this, therefore, the present study shows the presence of CoV infections in bats in Poland for the first time and additionally confirms the host restriction of some of these pathogens. SARS-related coronaviruses were detected in four bat roosts in southwestern Poland hosted by lesser horseshoe bats, whereas alphacoronaviruses were detected in *Myotis* spp. bats.

The range of the lesser horseshoe bat extends from western Europe to central Asia. The species has been regarded as endangered since the drastic decline of the horseshoe bat population in Germany, western France, and Poland in the 1960s. However, at the beginning of the 1990s its numbers stabilized and now are steadily rising. The occurrence of horseshoe bats in Poland (and specifically the lesser horseshoe bat, because only this species is found in Poland) is limited to the southern part of the country. During the summer they occupy warm attics of churches and other buildings, being close to humans, whereas winter roosts consist mostly of caves and mines [40]. Given the SARS-related coronaviruses’ specificity to the horseshoe bat, the rebounding population numbers of this rhinolophid and its proximity to human habitation for part of the year, the species merits continued research and surveillance.

The recently discovered SARS-CoV-2 in Wuhan revealed the closest relationship to viruses detected in material sampled from *Rhinolophus malayanus*, *R. affinis*, and Malayan pangolins (*Manis javanica*). Its full-genome similarity was estimated at 96.2% to a viral sequence reported in *R. affinis* (BatCoVRaTG13) originating from Yunnan province, China, and other SARS-rCoVs identified from pangolins also shared much identity with the Wuhan SARS-CoV-2 [10,11]. SARS-related CoV infections of lesser horseshoe bats in Europe were reported previously only in Italy, Slovenia, Spain, and Bulgaria and a new sarbecovirus was identified in the same bats in 2020 in the UK [31]. Here, we present evidence of the circulation of sarbecoviruses in Poland which extends the known geographical distribution of sarbecoviruses in Europe and additionally confirms their high host restriction to Rhinolophidae bats.

The prevalence of sarbecoviruses detected in Rhinolophus bat samples in Poland was estimated at 31.0%, whereas the overall detection rate of BtC oVs was estimated as 4.0% and was similar to the lower region of the range of BtCoVs prevalence in Europe which was between 1.4 and 21.0% [15,16]. The lower prevalence rate of BtCoVs in Poland in relation to the other European studies may have been caused by the quality of the fecal/guano samples, which contained many PCR inhibitors and sometimes required individualized preparative treatment. The differences in BtCoV prevalence in different European countries may be also the result of differing sensitivities of protocols and chemicals applied in the detection of BtCoVs and finally may derive from the particular bat species involved in the study as well as the number of tested samples. In Europe, alphacoronaviruses were mostly detected in *Myotis* (*M. daubentonii*, *M. dasycneme*, *M. nattereri*, and *M. bechsteinii*) and Pipistrelle (*P. pygmaeus*, *P. nathusii*, and *P. pipistrellus*) bat species, whereas betacoronaviruses were detected mostly in bats of the Rhinolophidae family and to a far smaller extent in Vespertilionidae [19,28,29,30,31,41]. In our study using a real-time RT-PCR method, sarbecoviruses were detected in 18 out of 58 bat samples collected from horseshoe bats in the Lower Silesia voivodeship inhabiting four independent roosts. The positive samples included 11 oral swabs taken in one roost twice in a one-year interval (May 2021 and May 2022) and 7 fecal samples including guano collected in another three bat roosts. The majority of positives were detected with very low Ct values (32–37.5 Ct), indicating a low coronaviral RNA load, and only five samples underwent successful Sanger sequencing. It is worth underlining that the samples with the highest viral loads for which Sanger sequencing was performed consisted of fecal samples. Coronaviral RNA was detected also in 13 oral swabs, however, with a low load. The prevalence of sarbecoviruses in Poland compared to other European countries was found to be high and at a similar rate as in Slovenia. In most European countries, the average prevalence of sarbecoviruses was estimated to range from 1.9 to 3.8% [29,31]. Such a high prevalence in bats in Poland can be explained by the active infection with BtCoVs in the tested bat roosts through the period of sampling and the permanent circulation of sarbecoviruses in the population of lesser horseshoe bats; circulation was a salient factor considering that all four infected roosts were in close proximity to each other, within a radius of approximately 20 km. The viral RNA was detected at the same roost after a one-year interval, and all sarbecoviruses sequenced in this study reached over 99.2% identity, which confirms the circulation of a homologous coronavirus in all infected bat roosts. The behavior of *R. hipposideros* lends weight to this speculation, because it is a sedentary species, and its migrations do not exceed 20 km in diameter [42]. The high prevalence refers to the previously observed seasonality of the detection of BtCoVs where the virus amplification was demonstrated to take place in maternity colonies [43]. However, to conclude, the general similarity of detected BtCoVs full genome sequencing and further studies will be performed. The amplified fragments corresponded to approximately 1.3% of the viral genome and allowed to draw phylogenetic relationships between detected BtCoVs.

While sarbecoviruses have been detected in horseshoe bats, alphacoronaviruses have been identified in *Myotis* genus members. Viral RNA was detected in two fecal samples collected from *M. daubentonii* bats in two roosts approximately 230 km from each other. They showed the highest phylogenetic relationship, of over 99% sequence identity, to BtCoVs identified in *Myotis daubentonii* in Denmark and the next highest to CoVs detected in *M. daubentonii* in Germany, Finland, and the UK based on the comparison of the 379 bp RdRp gene fragment. This specificity of CoVs to *M. daubentonii* observed in Poland bears out the previous observation of the host restriction of coronaviruses even over distances of greater than 600 km. Host restriction of CoVs was reported previously for both alpha and betacoronaviruses, particularly in *M. daubentonii* and *Rhinolophus ferrumequinum*, respectively, across various locations. This finding indicates that contact rates and seasonal movements of bats ensure efficient circulation and rapid diffusion of coronaviruses within the range of the host species [26]. *Myotis daubentonii* is a migratory bat species, usually travelling up to 150 km between summer and winter roosts. The longest documented overflight was 304 km. During swarming, *M. daubentonii* is the most numerous bat species in central and northern Europe. In a few roosts, especially in the lowlands, it forms large groups of up to 20,000 bats, and this facilitates contact between bats and the transmission of coronaviruses [42].

Coronaviruses are characterized by a high level of diversity, which is partially due to the infidelity of their polymerase and their atypically large genome. The high genetic diversity of CoVs facilitates their rapid adaptation to new hosts. The Polish BtCoVs revealed a high level of diversity between sarbecoviruses and alphacoronaviruses (identity of 60.93–61.2%), whereas within phylogenetic groups they conformed to a high level of identity (99.2–100% and 98.6% nucleotide sequence identity within sarbecoviruses and alphacoronaviruses, respectively). The BtCoVs detected in Poland clustered in phylogenetic groups highly restricted to the host and simultaneously distinct from coronaviruses circulating in other mammals (data not shown). We did not observe co-infections or interspecies jumps, although they may occur on rare occasions and particularly in a favorable ecological context such as when different species of bats co-roost. This promotes the spread of coronaviruses across the distribution area of the new host. Bayesian approaches revealed frequent cross-species transmission events occurring among bats which increased the risk of new CoVs emerging [37]. *Rhinolophus hipposideros* shares summer roosts with *Myotis emarginatus,* and because their distributions in Poland overlap, further studies screening sarbecoviruses in *M. emarginatus* would be highly valuable and are urgently needed. Jumping events were reported between mammals and bats, e.g., bat-to-rodent CoV transmission leading to the evolution of the distinct lineage A betacoronaviruses. An evolutionary study revealed that SARS-CoV-2 has required no significant adaptation to humans since the pandemic began, and that SARS-CoV-2 was able to be transmitted from human to human before it jumped from bats to humans [44].

In conclusion, our findings demonstrate that coronaviruses circulate among the bat population in Poland, confirm their high host specificity, and expand the data on the geographical distribution of coronaviruses, in particular sarbecoviruses, in Europe. They are highly diverse, comprising two genera, *Alpha*- and *Betacoronavirus*, the latter including sarbecoviruses, the SARS-related CoVs that were or are the etiological agents of recent epidemics. Coronaviruses, as zoonotic pathogens with the ability to cross the species barrier, pose a potential risk of virus transmission to animals and humans; however, the spillover mechanism of bat-related CoVs is multifactorial and mostly requires adaptation to replication in a new host. Due to anthropopressure and the nature and lifestyle of horseshoe bats, these bat–human contacts are becoming more frequent, and bats deprived of their natural habitat are increasingly establishing colonies in buildings occupied by people and their pets, in attics, lofts, and balconies in large cities, and in basements (personal observations). Therefore, strict bat coronavirus surveillance should be continued, particularly in the bat roosts where SARS-related CoVs were detected.

## Figures and Tables

**Table 1:** The prevalence of BtCoVs in different bat species included in the study. O—oral swabs; F—feces.

Species	No of Samples	No of Positives rtRT-qPCR	No of Positives nRT-PCR	Total No of Positives	Positives % (95% CI)
*Eptesicus serotinus*	57	0	0	0	0% (0.0–6.3)
*Nyctalus noctula*	79	0	0	0	0% (0.0–4.6)
*Nyctalus leisleri*	3	0	0	0	0% (0.0–56.1)
*Barbastella barbastellus*	55	0	0	0	0% (0.0–6.5)
*Plecotus auritus*	30	0	0	0	0% (0.0–11.4)
*Vespertillo murinus*	12	0	0	0	0% (0.0–24.2)
*Pipistrellus pygmaeus*	29	0	0	0	0% (0–11.7)
*Pipistrellus pipistrellus*	10	0	0	0	0% (0–27.8)
*Pipistrellus* spp.	6	0	0	0	0% (0.0–39.0)
*Pipistrellus nathusii*	19	0	0	0	0% (0.0–16.8)
*Myotis dasycneme*	3	0	0	0	0% (0.0–56.1)
*Myotis daubentonii*	55	0	2 (F)	2 (F)	3.6% (0.6–12.3)
*Myotis myotis*	4	0	0	0	0% (0–49.0)
*Myotis mystacinus*	6	0	0	0	0% (0–39.0)
*Myotis nattereri*	22	0	0	0	0% (0–14.9)
*Myotis bechsteinii*	8	0	0	0	0% (0–32.4)
*Myotis alcathoe*	1	0	0	0	0% (0–94.9)
*Myotis brandtii*	7	0	0	0	0% (0–35.4)
*Myotis emarginatus*	1	0	0	0	0% (0–94.9)
*Rhinolophus hipposideros*	58	18 (14 O; 4 F)	3 (F)	18 (14 O; 4 F)	31% (20.6–43.8)
unidentified	38	0	0	0	0% (0.0–9.2)
Total	503	20 (14 O; 6 F)			4.0% (2.6–6.1)

## Data Availability

The nucleotide sequences of BtCoVs detected in Poland were deposited in GenBank database with accession nos: ON873766, ON873768, ON873767, ON873769 and ON873770.
